# ReS_2_ Charge Trapping Synaptic Device for Face Recognition Application

**DOI:** 10.1186/s11671-019-3238-x

**Published:** 2020-01-03

**Authors:** Ze-Hui Fan, Min Zhang, Lu-Rong Gan, Lin Chen, Hao Zhu, Qing-Qing Sun, David Wei Zhang

**Affiliations:** State Key Laboratory of ASIC and System, School of Microelectronics, Fudan University, Shanghai, 200433 China

**Keywords:** Charge trapping memory, Synaptic device, Two-dimension material, Artificial neural network

## Abstract

Synaptic devices are necessary to meet the growing demand for the smarter and more efficient system. In this work, the anisotropic rhenium disulfide (ReS_2_) is used as a channel material to construct a synaptic device and successfully emulate the long-term potentiation/depression behavior. To demonstrate that our device can be used in a large-scale neural network system, 165 pictures from Yale Face database are selected for evaluation, of which 120 pictures are used for artificial neural network (ANN) training, and the remaining 45 pictures are used for ANN testing. A three-layer ANN containing more than 10^5^ weights is proposed for the face recognition task. Also 120 continuous modulated conductance states are selected to replace weights in our well-trained ANN. The results show that an excellent recognition rate of 100% is achieved with only 120 conductance states, which proves a high potential of our device in the artificial neural network field.

## Background

Since the advent of modern computers, the von Neumann structure, wherein the arithmetic unit is separated from the memory, has been widely used. This kind of structure makes data transmission between the arithmetic unit and memory becomes a bottleneck, significantly limiting the improvement of computer performances [1, 2]. Meanwhile, the arithmetic unit and main memory are both volatile devices with high energy consumption, and information will disappear immediately if the power is cut off [3]. In contrast, the human brain is an efficient information storage and computing system with high fault tolerance and low power consumption (about 20 W), and it is based on a highly interconnected, massively parallel, and structurally variable complex network consisted of about 10^11^ neurons and 10^15^ synapses [4, 5]. These neurons are considered to be the brain’s computational engines, receiving input signals from thousands of synapses in parallel. Synaptic plasticity is a biological process that changes synaptic weight through synaptic activities, and it is considered as a source of learning and memory [6].

**Fig. 1:**
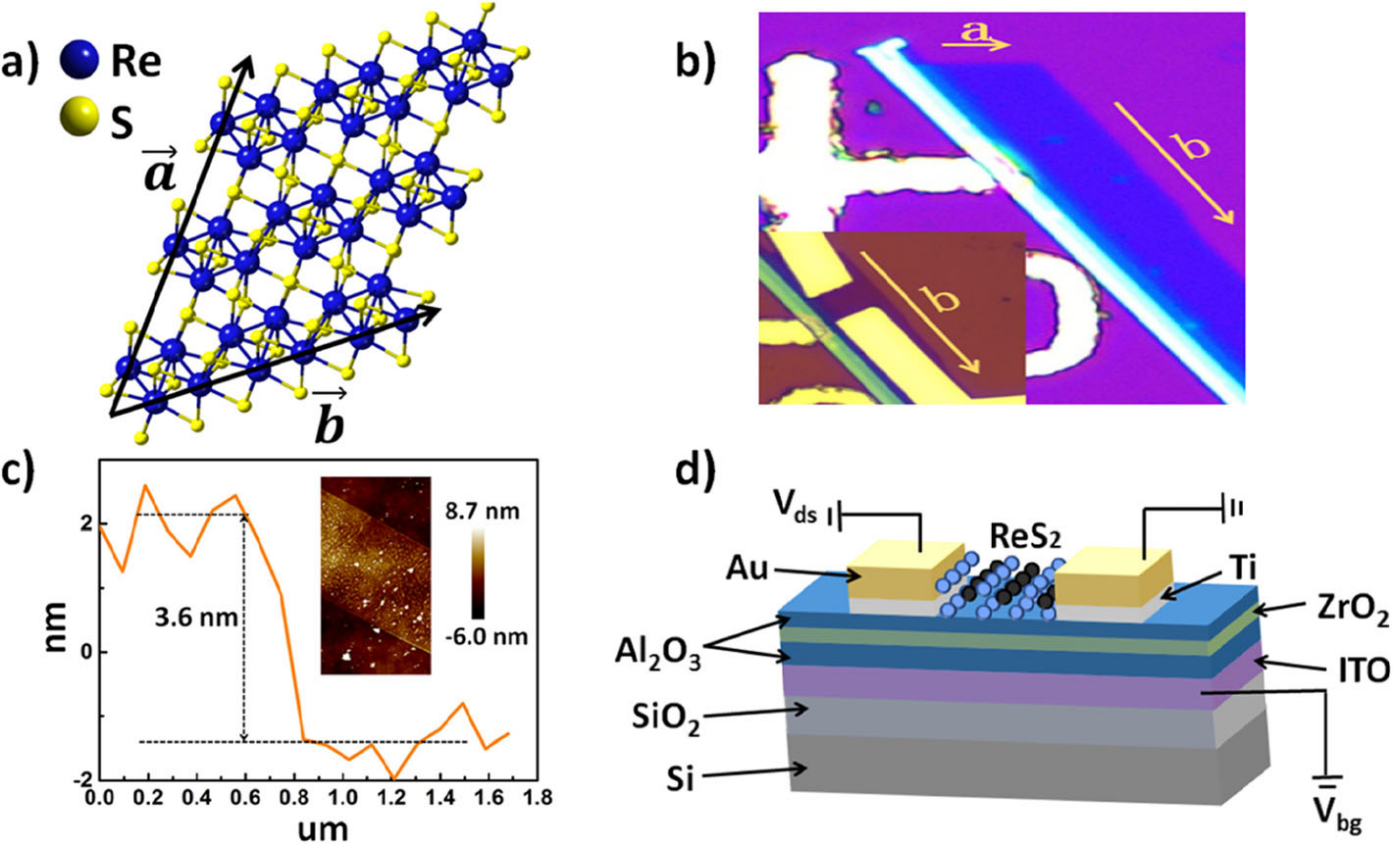
The synaptic devices based on ReS_2_ 2D material. **a** Crystal structure of monolayer ReS_2_. **b** Optical image of a five-layer ReS_2_ flake. Inset: source and drain electrodes patterned on the ReS_2_ flake; direction *b* is taken as the direction of channel current. **c** The AFM image and height profile of the ReS_2_ flake. **d** Schematic diagram of a 2D material ReS_2_ synaptic device. The thickness of the Al_2_O_3_, ZrO_2_, and Al_2_O_3_ stack (from bottom) is 12 nm, 4 nm, and 4 nm, respectively

The two-dimension (2D) materials with a small size and excellent electronic properties, such as graphene, transition metal dichalcogenides (TMDCs), and black phosphorus, have attracted significant attention and have been successfully implemented into synaptic devices [7, 8]. The TMDCs with the symmetric lattice, such as MoS_2_ and WSe_2_, have been widely studied [9, 10]. On the other hand, rhenium disulfide (ReS_2_) with a distorted octahedral (1T) crystal structure has been rarely explored in the neuromorphic field. Most TMDs have a direct bandgap in the monolayer and an indirect bandgap in the multilayer, so a monolayer material that is difficult to obtain is needed for good device performance. However, ReS_2_ within ten layers are all considered to have a direct bandgap [11], which means ReS_2_ within ten layers can all perform well. Besides, the asymmetric lattice structure leads to weaker interlayer coupling energy, which benefits the exfoliation work, and thus makes the synaptic device much easier to fabricate [12–15]. In this study, ReS_2_ film is used as a channel material. The crystal structure of monolayer ReS_2_ is shown in Fig. 1a, where directions *a* and *b* denote the second shortest axis and the shortest axis in the basal plane, respectively. Based on the previous scientific researches and plenty of optical images of our exfoliated ReS_2_ film [13], direction *b* denotes the crystallographic orientation with the highest electron mobility. To illustrate the electrical characteristics of our ReS_2_ synaptic device better, direction *b* is considered as a direction of channel current, as shown in Fig. 1b.

There have been many devices with different structures that successfully simulated synaptic dynamics, such as short-term plasticity (STP), long-term potentiation (LTP), and long-term depression (LTD) [16–18]. A MoS_2_/PTCDA hybrid heterojunction synapse has been demonstrated with efficient photoelectric dual modulation [10]. A carbon nanotube synapse [19] and silicon-based MoS_2_ synapse [20] showed dynamic logic. However, the mentioned studies focused only on the synaptic level. In some studies, different conductance states were realized to prove that their devices could be used to build artificial neural networks (ANNs), but they did not put the conductive states into the ANNs for calculation [21, 22]. In this work, 120 continuous conductance states are modulated, and the corresponding conductance values are used in the trained face recognition network for calculation; an excellent recognition rate of 100% is achieved.

## Methods

The schematic structure of our synaptic device is shown in Fig. 1d, where it can be seen that a 70-nm ITO (indium tin oxide) film was deposited on the SiO_2_/Si substrate as a back gate electrode. The substrate was a Si wafer with 200-nm SiO_2_ on top. It was first cleaned with the acetone, isopropyl alcohol, and deionized water, and then dried with N_2_ gas before the ITO deposition. The ITO layer was first deposited by sputtering and then annealed at 400 °C in the N_2_ atmosphere for 10 min by rapid thermal processing (RTP). Transparent ITO electrodes are used in order to accurately fabricate source and drain electrodes using electron beam lithography. The Al_2_O_3_/ZrO_2_/Al_2_O_3_ sandwiched structures with a thickness of 12 nm, 4 nm, and 4 nm were grown on the ITO by atomic layer deposition (ALD) as a barrier layer, an electron capture layer, and a tunneling layer respectively. Next, the mechanically exfoliated ReS_2_ flakes with a thickness of about 3.6 nm were deposited as a channel under the patterned Ti/Au electrodes. The Ti/Au electrodes with 10-nm and 70-nm thickness were patterned using the electron beam lithography followed by the electron beam evaporation as a source and a drain, respectively. Figure 1c shows the atomic force microscope image of our 3.6-nm thickness ReS_2_ film (about five layers); the channel length was designed to be 1.5 μm (see the inset in Fig. 1b). In this work, the ITO back gate acted as a presynapse neuron, and the Ti/Au electrodes acted as a postsynapse neuron. A small and constant voltage was applied between the source and drain electrodes, while the ITO back gate electrode was applied with pulses to modulate synaptic device performance.

## Results and Discussion

**Fig. 2:**
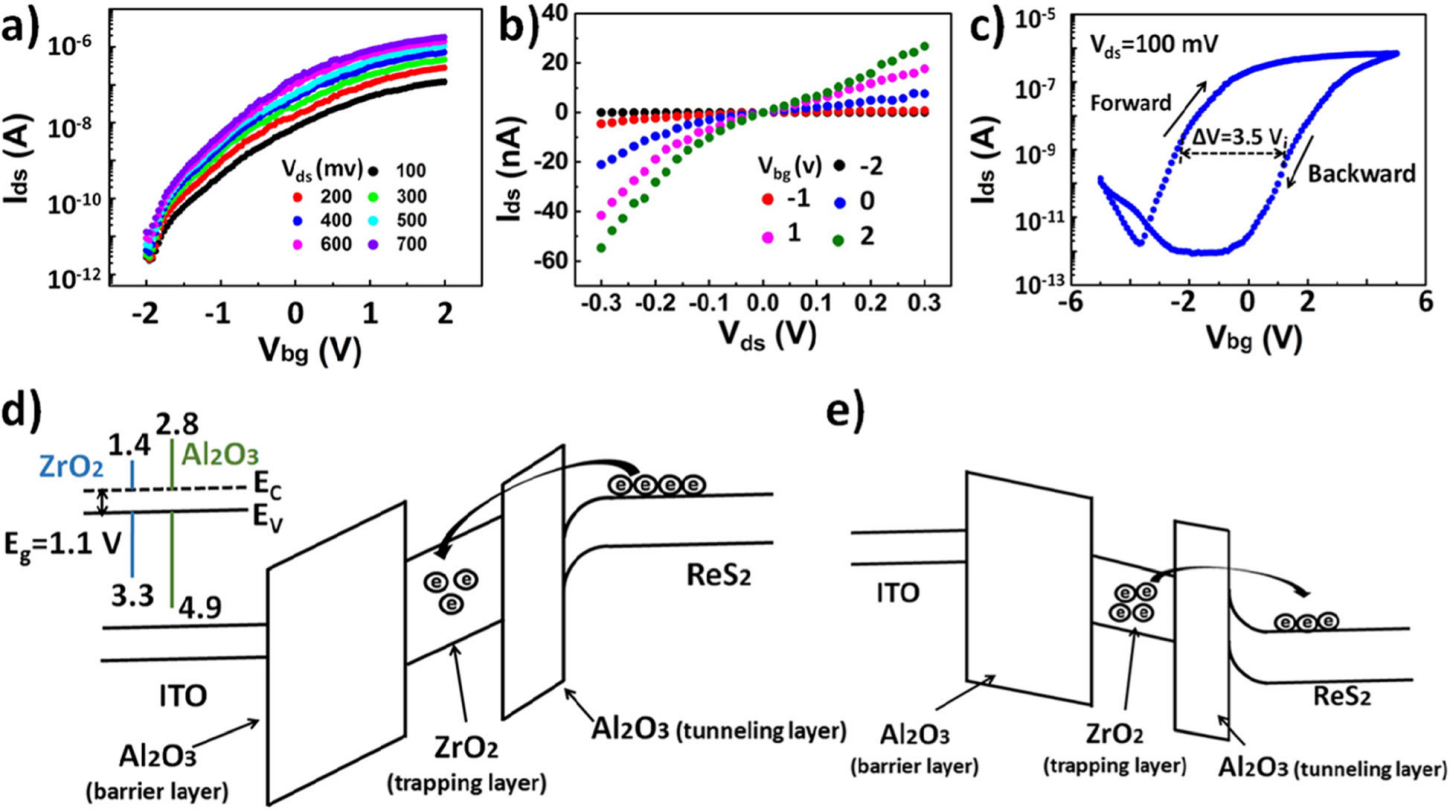
Electrical properties of the ReS_2_ synaptic devices. **a** Transfer characteristic (*I*_ds_–*V*_bg_) of the ReS_2_ synaptic devices at a fixed *V*_ds_ changing from 100 to 700 mV with the step of 100 mV. **b** Output characteristic (*I*_ds_–*V*_ds_) of the ReS_2_ synaptic devices at a fixed *V*_bg_ changing from − 2 to 2 V with the step of 1 V. **c** Hysteresis loop at *V*_bg_ of ± 5 V sweep ranges. *V*_ds_ was kept at 100 mV. **d** Energy band diagram of the ReS_2_ synaptic devices with positive back gate voltage. Inset: energy levels of Al_2_O_3_ and ZrO_2_. **e** Energy band diagram of the ReS_2_ synaptic devices with negative back gate voltage

Figure 2a shows the transfer characteristics of our synaptic device at a 2-V back gate voltage (*V*_bg_ = 2 V) under a fixed drain-to-source voltage (*V*_ds_) changing from 100 to 700 mV with the step of 100 mV. An On/Off current ratio over 10^6^ could be observed. The curve displayed the drain-to-source current (*I*_ds_), which first increased rapidly and then became saturated; the excellent saturation characteristics corresponded to the strong channel regulation by the ITO back gate electrode. Unlike the traditional transistors, which use silicon as a bottom gate electrode and SiO_2_ as a dielectric at the operation voltage of usually more than 20 V [23], the operation voltage of our synaptic device with only a 20-nm distance between the ReS_2_ channel and ITO back gate electrode was below 5 V, significantly improving the efficiency of synaptic device. The inset in Fig. 2a shows the superlinear relationship under the low-*V*_ds_ regimes, which demonstrates a good Schottky contact between the ReS_2_ channel and source and drain electrodes. As shown in Fig. 2b, *I*_ds_–*V*_bg_ hysteresis curve could be observed when *V*_bg_ changed from − 5 to 5 V and then reversed back at a constant bias of 0.1 V (*V*_ds_ = 0.1 V). In the measurements, a small constant voltage of 0.1 V was applied between the source and drain electrodes to “read” the postsynaptic current. The memory window, which provided the basis for synaptic performance, was about 3.5 V; such a big memory window made our ReS_2_ device very promising for synaptic applications [24]. Since the top of the valence band of ZrO_2_ was higher than that of Al_2_O_3_, and the bottom of the conduction band was lower than that of Al_2_O_3_ (see the inset in Fig. 2c), ZrO_2_ used as an intermediate layer sandwiched between alumina could capture charge effectively. The energy band diagrams under positive and negative back gate voltage are shown in Fig. 2c and d, respectively. When a positive voltage was applied, electrons in the ReS_2_ channel would first tunnel through the Al_2_O_3_ tunneling layer, then be captured by the ZrO_2_ trapping layer. On the contrary, when ITO was applied with a negative voltage, electrons gathered in the ZrO_2_ layer would be sent to the ReS_2_ channel; the energy bands bent in the direction of the channel.

**Fig. 3:**
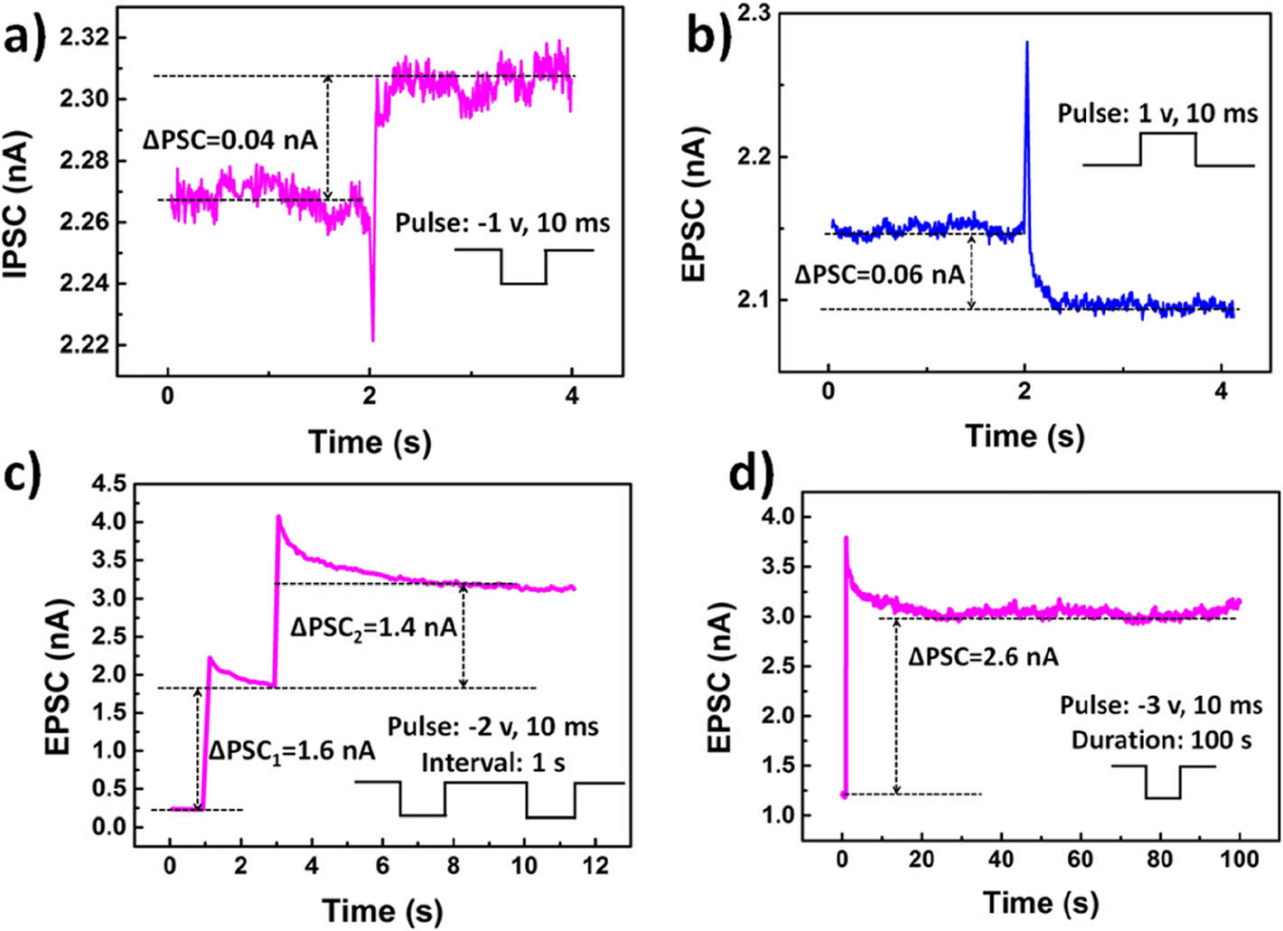
Synaptic performance of the ReS_2_ synaptic devices. **a** The excitatory postsynaptic current (EPSC) triggered by the input pulse (− 1 V, 10 ms). **b** The inhibitory postsynaptic current (IPSC) triggered by a presynaptic spike (1 V, 10 ms). **c** Pair of output spikes of EPSC triggered by two consecutive input pulses (− 2 V, 10 ms, and with a 1-s interval between pulses). **d** Retention characteristics of the ReS_2_ synaptic devices after a − 3 V and 10 ms presynaptic spike

In Fig. 3a, a typical excitatory postsynaptic current (EPSC) was detected after applying a negative input pulse (with the amplitude of − 1 V and duration of 10 ms) at the ITO back gate. Also, an inhibitory postsynaptic responded to a positive voltage pulse (with the amplitude of 1 V and duration of 10 ms) was observed in Fig. 3b, which is similar to a biological synapse [25]. The pulse signal from the presynapse neuron was transmitted to the postsynapse neuron through the synapse and converted into the postsynaptic current (PSC) [26]. The PSC value was determined by pulse amplitude and duration. When the pulse was negative, the electrons from the defects of ZrO_2_ gained enough energy to tunnel through the upper Al_2_O_3_ dielectric layer into the ReS_2_ channel. The constant value of the current was slightly higher than the previous value (∆PSC = 0.04 nA) and could maintain for a long time. This phenomenon corresponded to the long-term potentiation (LTP) in the biological synapse. However, when the pulse was positive, electrons in the ReS_2_ channel tunneled through the Al_2_O_3_ layer under the attraction of the electric field and were captured by the defects of ZrO_2_. Thus, the constant value of the current was slightly lower than the original value and could maintain the same for a long time (∆PSC = 0.06 nA). This process corresponded to the long-term depression (LTD) in the biological synapse. The LTP and LTD provided a physiological substrate for learning and memory in synaptic devices. When the negative pulses with the amplitude of − 2 V and duration of 10 ms were applied continuously, with a 1-s interval between pulses, the rising current in the two steps was observed, as shown in Fig. 3c. The rising current values were 1.6 nA and 1.4 nA, respectively. Therefore, a continuous and uniformly rising current could be obtained under the periodic gate voltage pulses, and the steady current after stimulation could last for a long time, as shown in Fig. 3d. This finding provided a basis for obtaining the multiple stable conductive states.

**Fig. 4:**
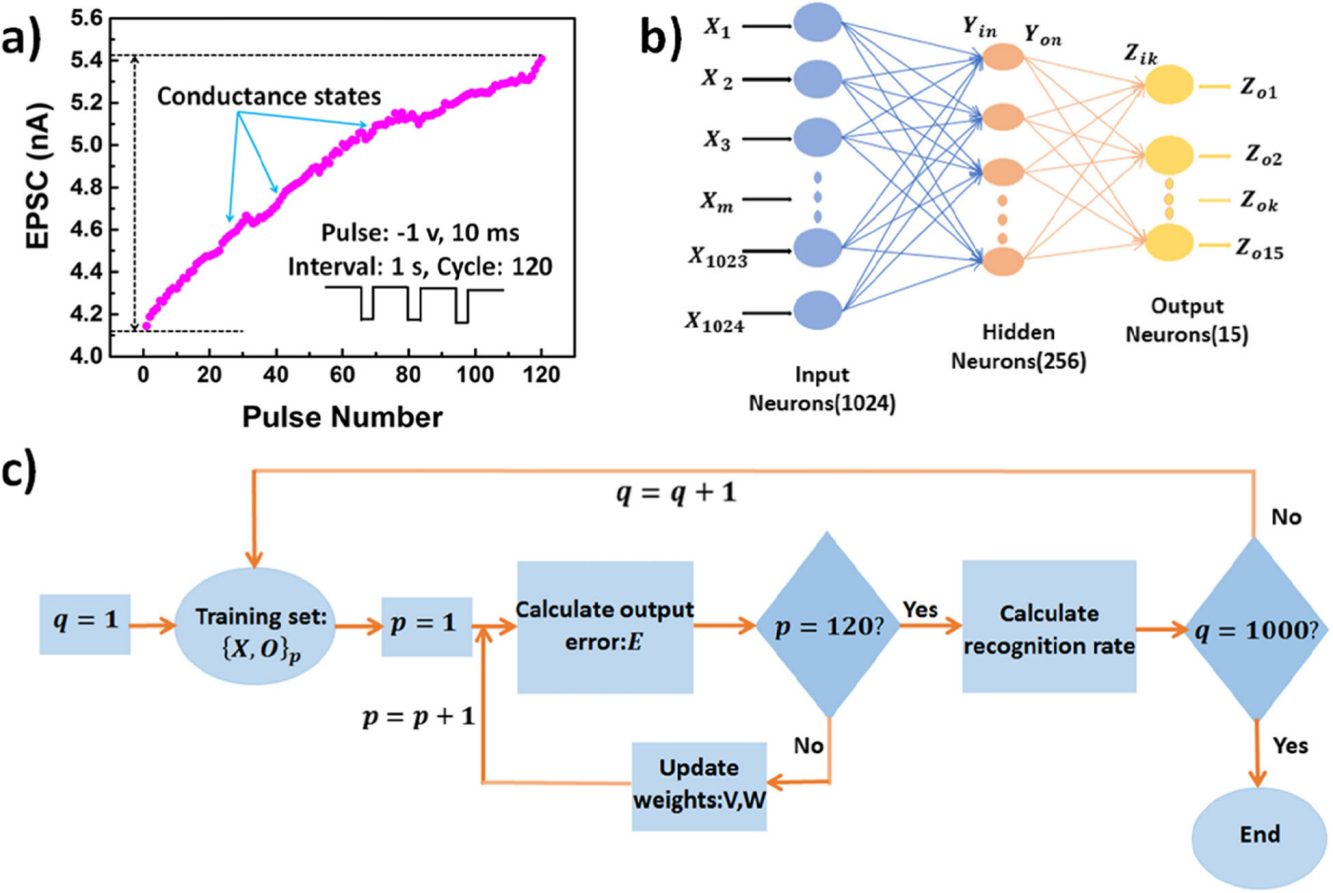
Artificial neural network for face recognition. **a** 120 conductance states after applying 120 negative pulses (− 2 V, 10 ms, and with a 1-s interval between pulses). **b** The three-layer ANN with 1024 input neurons, 256 hidden neurons, and 15 output neurons. **c** Flowchart of the training-recognition cycle

Figure 4a shows 120 current values after applying 120 negative pulses with an amplitude of − 2 V and a duration of 10 ms and with a 1-s interval between pulses. Apparently, the current curve showed excellent linearity, 120 effective high-stability conductance states were obtained in each state. Different conductance states corresponded to different ANN weight values [27].

In this work, a three-layer artificial neural network for face recognition task is proposed, and its structure is presented in Fig. 4b, wherein it can be seen that the input layer consists of 1024 neurons that correspond to 1024 pixels of an image, the middle (hidden) layer consists of 256 neurons, and the output layer consists of 15 neurons that correspond to 15 classes of faces.

The development of the proposed ANN is as follows. A total of 165 pictures, including 15 types of pictures from Yale Face database [28] are used for ANN training and testing. Eight images of each type are used for ANN training, and the remaining three images of each kind are used for ANN testing. Given that the modules are smooth functions relative to their inputs and their internal weights, the multilayer architectures can be trained by simple stochastic gradient descent, and the gradients are generally computed by the backpropagation procedure [29]. Therefore, we use the classical backward propagation (BP) algorithm to build our network and show how the BP algorithm works for our ANN.

In this work, *X*_*m*_ represents an input neuron, so the input value of a hidden neuron can be expressed as:

$$ {Y}_{in}=\sum \limits_{m=1}^{1024}{X}_m{V}_{mn} $$

where *V*_*mn*_ represents the weight value between an input neuron *X*_*m*_ and a hidden neuron *Y*_*in*_, and all *V*_*mn*_ form the matrix V having a total of 1024 × 256 weight values; the initial value of this matrix is randomly assigned. The activation function of the hidden layer is the sigmoid function, so the output value of a hidden neuron is given by:

$$ {Y}_{on}=\frac{1}{1+{e}^{Y_{in}}} $$

Thus, the input value of an output neuron can be expressed as:

$$ {Z}_{ik}=\sum \limits_{n=1}^{256}{Y}_{on}{W}_{nk} $$

where *W*_*nk*_ represents the weight value between a hidden neuron *Y*_*on*_ and an output neuron *Z*_*ik*_, and all *W*_*nk*_ form the matrix W with a total of 256 × 15 weight values; the initial value of *W*_*nk*_ is also randomly assigned. Besides, we use the sigmoid function as an activation function of the output layer, so that the output value of an output neuron is given by:

$$ {Z}_{ok}=\frac{1}{1+{e}^{Z_{ik}}} $$

Comparing the above-calculated output with the correct output, the total output error can be obtained, and it is expressed as:

$$ E=\frac{1}{2}\sum \limits_{k=1}^{15}{\left({O}_k-{Z}_k\right)}^2 $$

where *O*_*k*_ is the correct output value. So far, the forward propagation process of the network has been completely described. To improve the recognition rate, the backpropagation process is needed to calculate the errors of the weights, and they are used to update the network weights in the next iteration.

$$ \Delta  {V}_{mn}=\mu \frac{\partial E}{\partial {V}_{mn}} $$

$$ \Delta  {W}_{nk}=\mu \frac{\partial E}{\partial {W}_{nk}} $$

$$ {V_{mn}}^{\prime }={V}_{mn}+\Delta  {V}_{mn} $$

$$ {W_{nk}}^{\prime }={W}_{nk}+\Delta  {W}_{nk} $$

In the above mathematical expressions, *∆V*_*mn*_ and *∆W*_*nk*_ respectively represent the errors of *V*_*mn*_ and *W*_*nk*_; after adding the errors to the original weight, we get the updated weight *V*_*mn*_^′^ and *W*_*nk*_^′^; *μ* is the learning rate, and *μ* = 0.06. After updating the weights, a new image is fed to the ANN, and the weight update process is repeated until all 120 images have been used for training. Next, we use the trained network to identify the remaining 45 images and calculate the recognition rate. The ANN testing process requires only the forward propagation process. Each image used for testing gets 15 output values after a forward propagation. The output value reflects the probability that the input image is of a certain type. The output with the maximum probability value is selected, and the corresponding type is the type of the input picture identified by the network. The recognition results are compared with the standard output; all correctly identified pictures are counted, and their total number is *n*. In each training-recognition cycle, the recognition rate *r* is given by:

$$ r=\frac{n}{45}\times 100\% $$

Generally, the recognition rate of the first recognition is very low, and in our ANN with 256 hidden neurons, the first recognition rate is only 17.78%. The above training-recognition process is repeated until the maximum recognition rate is obtained. The whole training-recognition cycle is shown in Fig. 4c.

**Fig. 5:**
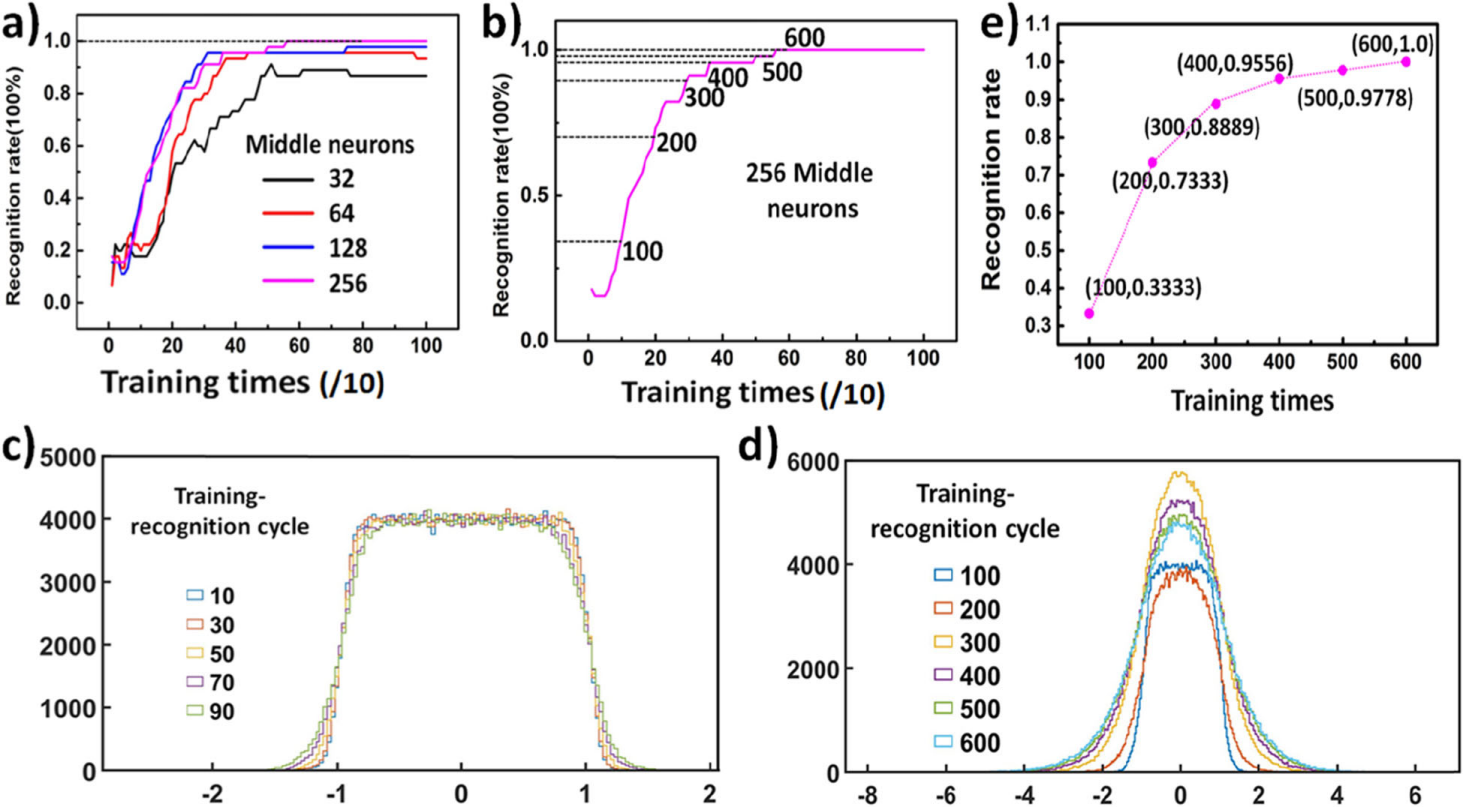
Realization of the face recognition. **a** Recognition rate curve at different numbers of hidden neurons (32, 64, 128, and 256). **b** Recognition rate curve at 256 hidden neurons; the recognition rate reaches 100% after nearly 600 training-testing epochs. **c** The distribution of weight values after 10 to 90 (in steps of 20) training epochs. **d** The distribution of weight values after 100 to 600 (in steps of 100) training epochs. **e** Recognition rate after the replacement; the weight values were replaced after 100~500 training epochs (in steps of 100)

As shown in Fig. 5a, during the ANN development process, the maximum recognition rate and rising speed of recognition rate (training speed) were different at a different number of hidden neurons. A larger number of hidden neurons led to a higher maximum recognition rate and a faster rising speed, but also increased energy consumption, so certain tradeoff should have to be made. In the case of 256 hidden neurons, the recognition rate reached 100% after 600 iterations of training, as shown in Fig. 5b. Since this was definitely the maximum recognition rate that could be achieved, in our ANN, we set the number of hidden neurons to 256. The distribution of weight values after different training-testing cycles is presented in Fig. 5c, and it indicates that the weights became more scattered after more cycles, that is to say, to reach a higher recognition rate, the weights in the ANN had to be adjusted. Once we achieved the maximum recognition rate, the matrices ***V*** and ***W*** having the optimal weight value were obtained. To demonstrate better that our ReS_2_ device is suitable to be applied to ANNs, all weight values in the weight matrices ***V*** and ***W*** were replaced by device’s conductance values. We used *I*_*j*_(*j* = 1, 2, 3⋯120) to represent 120 conductance values that were obtained after 120 cycles, and we made a linear transformation of the original conductance values so that conductance range was consistent with the weight range, which was given by:

$$ {C}_j=A{I}_j+B $$

where *C*_*j*_ represented the weight value after the linear transformation. In the case of 600 cycles, the linear transformation coefficients were *A* = 1.3769 × 10^10^ and *B* =  − 65.784. Next, we subtracted each *C*_*j*_ from each weight value and replaced the weight value with *C*_*j*_ that had the smallest absolute value after subtraction; namely, we calculated min|*V*_*mn*_ − *C*_*j*_|, min|*W*_*nk*_ − *C*_*j*_| and replaced each weight value with the corresponding *C*_*j*_. In this way, we obtained new ***V*** and ***W*** weight matrices wherein all the weight values were replaced by *C*_*n*_. Then, we used our new weight matrices in ANN testing, and the ANN recognition rate of 100% was achieved, which proved that our 120 conductance states could be perfectly used as weight values in the ANN. For the purpose of further analysis, we replaced the weight values after 100~500 training cycles (in steps of 100), and the identification results obtained after the replacement are completely consistent with the original one, as shown in Fig. 5d. This proves that these 120 current values could perfectly replace over 10^5^ weight values for calculation. By further increasing the number of gate pulses, more conductance states could be obtained, which proved that our ReS_2_ device could be used in a large-scale neural network system.

## Conclusions

In this work, we introduce a high-k dielectric stack based 2D ReS_2_ synaptic device and demonstrate some fundamental synaptic behaviors such as long-term potentiation and long-term depression. The results show that our ReS_2_ device can simulate synaptic performance well. Also, an ANN is constructed to prove the application of the proposed device in artificial neural networks. Applying 120 periodic gate voltage pulses, 120 effective, clearly distinguished conductance states are obtained, and they are used to replace more than 10^5^ weights in the ANN for face recognition. The recognition rate of 100% is achieved after replacement. This excellent result demonstrates that our ReS_2_ synapse can be used to build an artificial neural network.

## Data Availability

The authors declare that the materials, data, and associated protocols are available to the readers, and all the data used for the analysis are included in this article.

## Abbreviations

2D: Two-dimension
ALD: Atomic layer deposition
ANN: Artificial neural network
LTD: Long-term depression
LTP: Long-term potentiation
